# DNA replication recruits a friend to overcome a challenging break-up

**DOI:** 10.1038/s44318-024-00204-3

**Published:** 2024-08-21

**Authors:** James M Dewar

**Affiliations:** Vanderbilt University School of Medicine, Nashville, TN USA

**Keywords:** DNA Replication, Recombination & Repair

## Abstract

Two recent articles reveal that leftover replisome impede the next round of replication, and how specific helicases can help to overcome this problem.

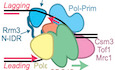

## Teaser

Replication complexes, or replisomes, are removed from DNA upon completion of DNA synthesis, but the importance of replisome removal, as well as how cells might respond to defects in this process, has remained elusive. New work by Polo Rivera et al (2024) and Olson et al (2024) reveals that leftover replisomes are an obstacle to replication and provides a first glimpse into the biochemical mechanisms that solve this problem.

**Figure 1:**
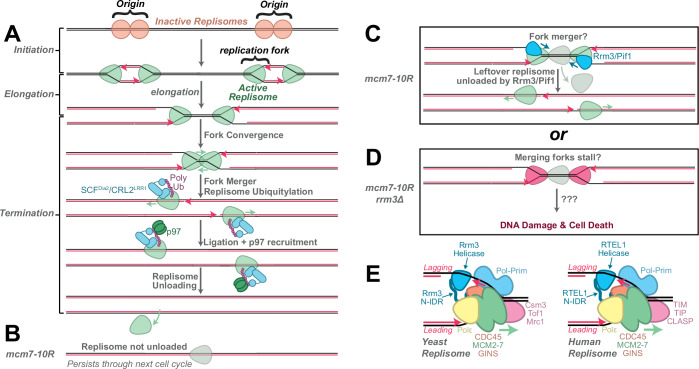
Mechanisms of replication termination. (**A**) Cartoon depicting the three main stages of DNA replication: initiation, elongation, and termination. Key steps of termination are shown in detail, except for resolution of double-stranded DNA intertwines by topoisomerases, omitted for simplicity. (**B**) Cartoon depicting retention of replisomes in the *mcm7-10R* mutant, described by Polo Rivera et al (**C**) Cartoon depicting the likely mechanism through which leftover replisomes are removed by Rrm3/Pif1 in the *mcm7-10R* mutant, based on the results of Polo Rivera et al (**D**) Cartoon depicting the consequences of defective replisome removal in the *mcm7-10R rrm3Δ* mutant, based on the results of Polo Rivera et al (**E**) Cartoon depicting the structures of the human and yeast replisomes as well as the recruitment mechanisms for Rrm3 and RTEL1 identified by Olson et al.

In eukaryotes, faithful DNA replication is enforced by the fidelity and spatiotemporal regulation of the replication machinery (“replisome”). In humans, ~60,000 pairs of replisomes are loaded onto DNA, activated (Fig. 1A, “initiation”), and then diverge from the site of loading (Fig. 1A, “origins”) to synthesize kilobases of DNA (Fig. 1A, “elongation”). Replisomes emanating from adjacent origins ultimately converge on the same stretch of DNA, resulting in “termination” (Fig. 1A), which is historically the least-studied stage of DNA replication. Converging replisomes can seamlessly pass each other because they encircle different strands of the parental DNA (Fig. 1A, “fork merger”) (Dewar et al, 2015; Dewar and Walter, 2017). The replisome is then removed from DNA (Fig. 1A, “replisome unloading”), which involves ubiquitylation of the replisome by a ubiquitin ligase (SCF^Dia2^ in yeast, CRL2^Lrr1^ in vertebrates), followed by removal of the replisome from DNA by the p97 ATPase. Defects in the fork-merger stage of termination were reported several decades ago in prokaryotic systems and more recently in eukaryotic systems (Campos et al, 2023; Deegan et al, 2019). In contrast, the need for a replisome unloading mechanism has remained unclear.

After replisomes pass each other during termination, they translocate along the template DNA strand until they encounter, and then slide over, double-stranded DNA from the downstream lagging strands of the opposing fork (Fig. 1A). Accordingly, complete synthesis and ligation of DNA occur in *Xenopus* egg extracts regardless of whether replisome unloading takes place (Dewar et al, 2015). Similarly, yeast cells lacking SCF^Dia2^ are unable to ubiquitylate and unload the replisome, but do not exhibit any delay in the completion of S phase or mitosis (Maric et al, 2014). It was therefore conceivable that replisome unloading was not important for DNA replication. However, although SCF^Dia2^ and CRL2^Lrr1^ are evolutionarily unrelated, they share the same mechanism of replisome recognition and thus represent an example of convergent evolution (Jenkyn-Bedford et al, 2021); this suggested that replisome unloading may be required in both yeast and vertebrates as a solution for an evolutionarily conserved obstacle to DNA replication.

Yeast *dia2∆* cells lacking SCF^Dia2^ activity exhibit genome instability, sensitivity to DNA damaging agents, and growth defects. It was not previously possible to attribute these phenotypes to defects in replisome unloading, because SCF^Dia2^ has various substrates. Also, while human cells lacking CRL2^Lrr1^ exhibit slowed DNA synthesis due to defects in replisome recycling (Fan et al, 2021), similar defects are not seen in yeast cells lacking SCF^Dia2^ (Maric et al, 2014) and could thus also reflect other targets of CRL2^LRR1^. To address the role and importance of replisome unloading, Polo Rivera et al (2024) created a ubiquitylation-deficient allele of *MCM7*, the primary replisome component that is ubiquitylated by SCF^Dia2^. The resulting *mcm7-10R* mutant is defective for ubiquitylation and replisome unloading both in reconstituted reactions as well as in cells (Fig. 1B). Although *mcm7-10R* mutants still underwent residual replisome ubiquitylation and unloading, Polo Rivera et al found that combining this allele with the *dia2-∆TPR* allele, which inhibits recruitment of SCF^Dia2^ to the replisome, severely inhibited replisome unloading, thus providing a genetic tool to examine the consequences of replisome unloading defects. Strikingly, the *mcm7-10R dia2-∆TPR* double mutants phenocopied full *dia2∆* mutants, demonstrating that the genome instability and growth defects in *dia2∆* cells were mostly attributable to replisome unloading defects. These experiments are the first to show a direct role for replisome unloading in ensuring genome stability.

Analysis of genome stability in *mcm7-10R* mutants by Polo Rivera et al (2024) led to a surprising discovery; *mcm7-10R* mutants experienced spontaneous DNA damage that was greatest during the *second* cell cycle after replisomes were allowed to accumulate on DNA. This observation suggested that replisome retention created a problem in the ensuing cell cycle. The 5’−3’ helicases Rrm3 and Pif1 are important for facilitating fork merger during termination (Deegan et al, 2019), and Polo Rivera et al noted that null mutants also exhibit synthetic lethality with *dia2∆* mutants, suggesting that Rrm3 and Pif1 might respond to defects in replisome unloading. The authors found that *mcm7-10R rrm3∆* mutants, but not *mcm7-10R pif1∆* mutants, were synthetically sick while *mcm7-10R rrm3∆ pif1∆* mutants were synthetically lethal. These results indicated that Rrm3 plays an important role when replisome unloading is defective, and Pif1 can partially compensate for this role. Strikingly, *mcm7-10R rrm3∆* mutants exhibited a stronger replisome-unloading defect and increased DNA damage compared to *mcm7-10R* mutants. This indicated that Rrm3 acts to limit DNA damage by promoting the unloading of non-ubiquitylated replisomes. Elegant cell-cycle synchronization experiments by Polo Rivera et al showed that Rrm3 acts in the second S phase to remove replisomes that persist from the first S phase. Overall, these data show that replisomes persisting from the previous cell cycle can create DNA damage during S phase if not removed by Rrm3 and, most likely, also Pif1.

It is unclear how Pif1 and Rrm3 remove leftover replisomes, but previous studies suggest a simple mechanism. Pif1 and Rrm3 unwind DNA ahead of replication forks, and Pif1 helicase has been reported to displace inactive replisomes from DNA (preprint: Hill et al, 2020). Thus, the simplest model consistent with the data by Polo Rivera et al (2024) is that Rrm3 and Pif1 unwind DNA to remove leftover replisomes from the prior cell cycle (Fig. 1C). Replisomes normally pass each other during termination because they encircle opposite DNA strands (Dewar et al, 2015). In contrast, replisomes encircle both DNA strands after termination, which would prevent them from being bypassed. The core replisome components can slide on DNA (Gros et al, 2015), suggesting that a retained replisome will probably slide ahead of a new replisome until an opposing replisome is encountered during termination (Fig. 1D). At this point it is likely that Rrm3 and Pif1 would unwind the DNA between converging replisomes to remove the retained replisome. However, it was also reported that a nucleosome can block the core replisome components from sliding on DNA (preprint: Hill et al, 2020) so it will be important to directly test this model.

Polo Rivera et al (2024) identify a new role for the 5’−3’ helicases Rrm3 and Pif1 during termination, in addition to their previously described role in facilitating fork merger. These enzymes can directly recognize DNA, but it is challenging to envisage how direct recognition of DNA substrates would allow for seamless termination, without slowing or stalling, as previously reported (Dewar et al, 2015). To address this question, Olson et al (2024) employ a tour de force of structure-function analyses. Olson et al identify an intrinsically disordered region (IDR) in the N-terminal region of Rrm3 and use both biochemical reconstitution and genetic experiments to show that this ‘N-IDR’ is necessary for Rrm3 function during termination. N-IDR is required for recruitment of Rrm3 to the replisome and, strikingly, fusion of N-IDR to an unrelated helicase with the same polarity supports termination. Thus, the N-IDR physically links 5’−3’ helicase activity to the replisome to ensure efficient termination. A combination of Alphafold modeling, biochemical reconstitution, and genetic analysis identified interactions between N-IDR and the replisome components Polε and GINS that are crucial for Rrm3 recruitment to the replisome and function during termination (Fig. 1E). Thus, Olson et al reveal the protein-protein interactions between Rrm3 and the replisome that allow Rrm3 to function during termination. To address whether this is conserved beyond yeast, Olson et al (2024) performed Alphafold modeling of vertebrate 5’−3’ helicases. They identified an N-IDR in RTEL1, a helicase implicated in replication termination (Campos et al, 2023), and showed that the N-IDR allows RTEL1 to interact with POLε within the vertebrate replisome (Fig. 1E). Thus, the N-IDR recruitment mechanism identified for yeast Rrm3 appears to be evolutionarily conserved in vertebrates.

The results of Olson et al point to an elegant mechanism with an exciting implication. The IDR of both Rrm3 and RTEL1 contacts the leading strand polymerase, and the N-IDR length is ideally suited to allow Rrm3/Rtel1 to access the lagging strand DNA template, upon which it would need to translocate in order to unwind DNA ahead of the replication fork (Fig. 1E). Furthermore, Olson et al note that Rrm3 and RTEL1 are evolutionarily unrelated yet both function during termination and share a recruitment mechanism. This represents yet another example of convergent evolution within the termination apparatus, just like SCF^Dia2^ and CRL2^LRR1^, which are also evolutionarily unrelated but nevertheless interact with the replisome using a conserved biochemical mechanism. The recruitment mechanism of Rrm3/RTEL1 establishes convergent evolution as a paradigm within the termination machinery and cements the view that the obstacles to termination are evolutionarily conserved, but the solutions were developed relatively late during evolution.

The results of these studies raise important future directions. The Polo Rivera et al study lays the foundation for understanding the cellular consequences of termination defects. Up until this point it has been challenging to specifically interfere with termination because many of the proteins involved play roles during other aspects of replication. For example, Rrm3 responds to replisome stalling at protein barriers (Brüning et al, 2014) and CRL2^Lrr1^ is crucial for fork progression by ensuring replisome recycling (Fan et al, 2021). However, it should now be possible to deliberately and specifically examine the consequences of defects in the final stages of DNA replication by comparison of *mcm7-10R and MCM7* strains (perhaps in a *rrm3∆* background to exacerbate the effect). Olson et al have raised important questions about the functions of 5’−3’ helicases during termination. Rrm3 appears to be constitutively associated with the replisome (Reusswig et al, 2022), while RTEL1 recruitment is regulated (Campos et al, 2023). This difference suggests that constitutive recruitment of RTEL1 to replisomes may be problematic in a way that constitutive Rrm3 recruitment is not, perhaps due to the susceptibility of the vertebrate replisome to the generation of lagging strand gaps (Zellweger et al, 2015) that would be substrates for RTEL1. It will be important to understand the importance of regulated, rather than constitutive, recruitment of RTEL1 to the replisome and the underlying mechanisms of regulation.

In conclusion, Polo Rivera et al (2024) and Olson et al (2024) reveal key mechanisms of replication termination and provide genetic tools to dissect this under-studied aspect of DNA replication. These works are a testament to the awesome power of yeast genetics and highlight the value of combining classical genetic approaches with cutting-edge biochemical reconstitution.
